# Association between Caffeine Intake and All-Cause and Cause-Specific Mortality: An Analysis of the National Health and Nutrition Examination Survey (NHANES) 1999–2014 Database

**DOI:** 10.3390/nursrep11040083

**Published:** 2021-11-10

**Authors:** Juan Feng, Jing Wang, Mini Jose, Yaewon Seo, Li Feng, Song Ge

**Affiliations:** 1 School of Nursing, University of Texas Medical Branch, Galveston, TX 77555, USA; mmjose@utmb.edu; 2 College of Nursing and Health Innovation, University of Texas at Arlington, Arlington, TX 76010, USA; jing.wang@uta.edu (J.W.); yaewon.seo@uta.edu (Y.S.); 3 Mathematics, Computer Science and Physics, Capital University, Columbus, OH 43209, USA; lfeng@capital.edu; 4 Department of Natural Sciences/Nursing, University of Houston Downtown, Houston, TX 77002, USA; ges@uhd.edu

**Keywords:** caffeine, coffee, cardiovascular mortality, all-cause mortality, cancer mortality

## Abstract

Sixty-four percent of adults in America drink coffee daily, and caffeine is the main reason people tend to drink coffee habitually. Few studies have examined the association between caffeine and all-cause and cause-specific mortality. The objective of this study was to examine the association between caffeine and all-cause and cause-specific mortality using the National Health and Nutrition Examination Survey (NHANES) 1999–2014 database. The multivariate Cox proportional hazards regression model was used to examine 23,878 individuals 20 years and older. Daily caffeine intake was measured once at baseline. A total of 2206 deaths occurred, including 394 cardiovascular (CVD) deaths and 525 cancer deaths. Compared to those with a caffeine intake of <100 mg/day, the hazard ratios (HRs) for CVD mortality were significantly lower in the participants with a caffeine intake of 100–200 mg/day (HR, 0.63; 95% confidence interval [CI], 0.45–0.88), and those with a caffeine intake of >200 mg/day (HR, 0.67; 95% CI, 0.50–0.88) after adjusting for potential confounders. The HRs for all-cause mortality were significantly lower in the participants with a caffeine intake of 100–200 mg/day (HR, 0.78; 95% CI, 0.67–0.91), and those with a caffeine intake of >200 mg/day (HR, 0.68; 95% CI, 0.60–0.78). Subgroup analyses showed that caffeine may have different effects on all-cause mortality among different age and body mass index (BMI) groups. In conclusion, higher caffeine intake was associated with lower all-cause and CVD mortality.

## 1. Introduction

Sixty-four percent of adults in America drink coffee daily, and the average coffee intake is 3.1 cups/day [1]. Traditionally, people were recommended to avoid or reduce coffee, especially those with a history of cardiovascular disease (CVD), because it increases blood pressure (BP), total cholesterol, low-density lipoprotein (LDL) cholesterol, and triglycerides [2]. More recent studies have reported that coffee improves insulin sensitivity, reduces chronic inflammation and liver enzymes, and may be inversely associated with all-cause and some of the cause-specific mortality rates [3,4,5]. A recent meta-analysis [6] pooled 40 studies with 3,852,651 participants and reported that the lowest hazard ratio (HR) was an intake of 2.5 cups of coffee per day for CVD mortality (HR, 0.83; 95% confidence interval [CI], 0.80–0.87; *p* < 0.001), and 3.5 cups/day for all-cause mortality (HR, 0.85; 95% CI, 0.82–0.89; *p* < 0.001).

Coffee is the main source of caffeine in the American diet, providing 71% of caffeine intake [7]. Caffeine can also be provided by tea, soda, energy drinks, chocolate, and cocoa-containing products [8]. Americans consume about 165 mg of caffeine per person per day [9]. Most previous studies have examined the association between coffee and mortality; only one study examined the association between caffeine and mortality and found no association with CVD mortality [10].

In the current study, we examined the association between caffeine intake and all-cause, CVD, and cancer mortality during 16 years of follow-up among 23,878 participants in the National Health and Nutrition Examination Survey (NHANES) 1999–2014 database.

## 2. Materials and Methods

**Figure 1:**
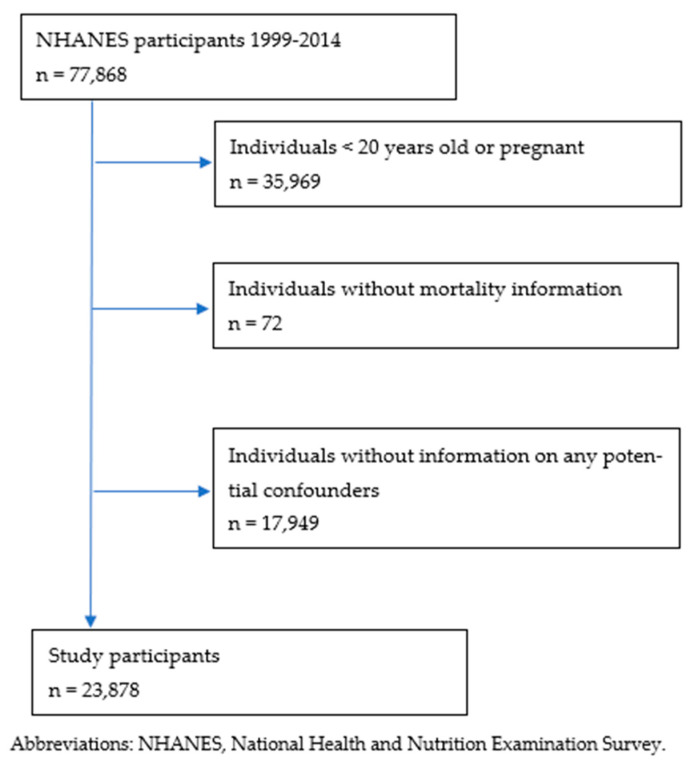
Participant flowchart.

Secondary data analysis was conducted using the NHANES 1999–2014 database. The NHANES is a periodic survey conducted by the National Center for Health Statistics (NCHS) of the Centers for Disease Control and Prevention (CDC). It is a national effort to assess the health and nutritional status of children and adults in the US [11]. NHANES used a stratified multistage probability sampling design to enable the representation of the non-institutionalized civilian US population [11]. The NHANES participants completed a structured interview at home and a physical examination at the mobile examination centers (MECs). Adults aged 20 years or older (who are not pregnant) with caffeine consumption information were included. Participants with missing information on any potential confounders were excluded (Figure 1). The current study examined data from 23,878 NHANES participants aged 20 to 85 years at baseline.

For all the NHANES participants, a 24-h dietary recall interview was administered in person during the examination in a private room at the MECs. The US Department of Agriculture (USDA) and the Department of Health and Human Services (DHHS) partnered to conduct the dietary interview, which was sent electronically from the field and imported into Survey Net (a computer-assisted food coding and data management system developed by the USDA) [12]. The participants were provided a standard set of measuring guides (measuring cups, spoons, and a ruler) and a food model booklet to help them report the volume and dimensions of the food items consumed during the 24-h period before the interview. The USDA designed a dietary data collection instrument: the Automated Multiple-Pass Method (AMPM), to provide an efficient and accurate way to collect intake data for large-scale national surveys.

During the 2003–2014 cycles, the NHANES conducted a second dietary interview by telephone 3 to 10 days after the first interview but not on the same day of the week to obtain a complete picture of the dietary patterns. The mean of the nutritional information from both recalls during these cycles was used in this study. Caffeine intake in the 24-h period was estimated by using the USDA food and nutrient databases for dietary studies (FNDDS) 5.0 [13] and available in the NHANES database. The USDA National Nutrient Database for Standard Reference provides the basis of nutrient values for foods and beverages [13]. The sources of nutrient data for this database include data provided by the food companies and trade associations, USDA analytical contracts, and literature [13].

Caffeine intake was calculated including these sources: coffee, tea, soda, energy drinks, chocolate, and cocoa-containing products [8]. Considering each cup (8 oz/240 mL) of ground roasted coffee contains about 96 mg of caffeine [14], the daily intake of caffeine was divided into three categories (<100 mg/day, 100–200 mg/day, and >200 mg/day).

The mortality data came from the 2015 public-use Linked Mortality Files (LMF), which are available for NHANES participants for the period of 1999–2014 and have been updated through 31 December 2015 [15]. The survival time was determined by the number of person-months of follow-up from NHANES interview date to the end of the mortality period, 31 December 2015. NHANES used the International Classification of Diseases, Tenth Revision (ICD-10) for deaths that occurred in or after 1999. In the 2015 public-use LMF, the codes that are used for CVD mortality include acute rheumatic fever (I00-I02), chronic rheumatic heart diseases (I05-I09), hypertensive heart disease (I11), hypertensive heart and chronic kidney disease (I13), ischemic heart diseases (I20-I25), pulmonary heart disease and disease of pulmonary circulation, acute pericarditis, endocarditis, valvular disorders, cardiomyopathy, cardiac arrest, atrial fibrillation, and heart failure (I30-I51). This study focused on all the CVD deaths and excluded stroke deaths because stroke ranks fifth among all causes of death behind heart disease, cancer, chronic lower respiratory disease, and accidents [16], and the mortality rate for stroke is usually listed separately in statistic reports. The codes used for cancer mortality include C00-C97: malignant neoplasms of lip, oral cavity, pharynx, digestive organs, respiratory and intrathoracic organs, bone and articular cartilage, mesothelial and soft tissue, breast, female and male genital organs, urinary tract, eye, brain and other parts of the central nervous system, thyroid and other endocrine glands, lymphoid, hematopoietic and related tissue, ill-defined, other secondary and unspecified sites, malignant and secondary neuroendocrine tumors, melanoma and other malignant neoplasms of the skin.

The demographic variables included age, sex, race, education, income, personal history of hypertension (HTN), diabetes, and cancer at baseline, smoking status, body mass index (BMI), and total daily intake of energy, carbohydrates, fat, and protein. These were obtained from NHANES questionnaires, interviews, and physical examinations. Hypertension (HTN) is defined as either a previous diagnosis of HTN or intake of antihypertensive medications. Diabetes is defined as either a previous diagnosis of diabetes or an HbA1c level of ≥6.5% or intake of antidiabetic medications, including insulin. Cancer is defined as a previous diagnosis of cancer. The BMI was calculated as body weight in kilograms divided by height in meters squared.

Race was classified into four categories: black, Hispanic, white, and other. Education was classified into four categories: <high school, high school, some college, and postgraduate. Income was classified into six categories: <15,000, 15,000–25,000, 25,000–35,000, 35,000–55,000, 55,000–75,000, and >75,000 dollars/year. Smoking status was divided into two categories: smokers and non-smokers. BMI was classified into four categories: underweight (<18.5 kg/m^2^), normal (18.5–24.9), overweight (25–29.9), and obese (≥30). Daily intake of total energy was presented as kilocalories (kcal). Daily intake of carbohydrates, fat, and protein were presented as grams per 100 kcal.

### Statistical Analysis

All data were entered into SAS statistical software, version 9.4 (SAS Institute Inc., Cary, NC, USA), and checked for missing data, which was excluded from the analysis. Demographic data were presented as numbers and percentages for categorical variables or mean ± standard deviation (SD) for continuous variables. We fitted the multivariate Cox proportional hazards model to the NHANES data to measure the effect of caffeine intake on hazards of all-cause mortality, CVD mortality, and cancer mortality, controlling for potential confounders age, sex, race, education, income, smoking status, BMI, total daily intake of energy, carbohydrates, fat, and protein, and presence of HTN, diabetes, and cancer at baseline. One of the fundamental assumptions of the Cox model is proportional hazards, which assume the effect of a factor is constant over time. When this assumption is violated, that means the effect of the factor might be changing over time. Thus, it would be worth including the interaction of the factor and survival time. The HRs and 95% CIs for mortality from all-cause, CVD, and cancer in participants with a caffeine intake of 100–200 mg/day and >200 mg/day were compared with those having a caffeine intake of <100 mg/day. Because of the scale difference in variables, the values of total energy were divided by 100; the values of carbohydrates, protein, and fat were divided by 10. Statistical tests were two-sided, and *p* < 0.05 was considered statistically significant for all tests.

## 3. Results

Table 1 presents the baseline characteristics of participants stratified by daily caffeine intake. Among the 23,878 participants, 12,006 (50.3%) had a caffeine intake of <100 mg/day, 5624 (23.6%) had a caffeine intake of 100–200 mg/day, and 6248 (26.1%) had a caffeine intake of >200 mg/day. Higher caffeine consumers were more likely to be older, female, white, smokers, have a higher BMI, a higher income, and a higher education level; they were more likely to have a higher daily intake of total energy, carbohydrates, protein, and fat; they were less likely to report a history of HTN and diabetes, more likely to report a history of cancer with *p* values < 0.05 across all categories.

During 16 years of follow-up, a total of 2206 deaths occurred, including 394 cases of CVD death and 525 cases of cancer death. Table 2 shows the HRs and 95% CIs for the effects of caffeine consumption on all-cause, CVD, and cancer mortality adjusted for age, race, sex, education, income, BMI, smoking status, total daily intake of energy, carbohydrates, protein, and fat, and the presence of diabetes, HTN, and cancer at baseline. Compared to those with a caffeine intake of <100 mg/day, the HRs for CVD mortality were significantly lower in the participants with a caffeine intake of 100–200 mg/day (HR, 0.63; 95% CI, 0.45–0.88), and those with a caffeine intake of >200 mg/day (HR, 0.67; 95% CI, 0.50–0.88) after multivariate adjustment (*p* < 0.05 for trend). Compared to those with a caffeine intake of <100 mg/day, the HRs for all-cause mortality were significantly lower in the participants with a caffeine intake of 100–200 mg/day (HR, 0.78; 95% CI, 0.67–0.91), and those with a caffeine intake of >200 mg/day (HR, 0.68; 95% CI, 0.60–0.78) after multivariate adjustment (*p* < 0.001 for trend). There was no association between caffeine intake and cancer mortality.

We tested the proportionality assumption using the standard approach by including a time-dependent function of the interaction between each factor and log((time+0.1)/87), where 0.1 was added to the logarithm function of the survival time to avoid evaluating the logarithm of very small values, and 87 was the median survival time. The chi-square test was not significant for each factor except age (*p* = 0.036) for all-cause mortality, i.e., the age effects were not proportional over time for all-cause mortality only. This test supported the need for stratifying the Cox model by age for all-cause mortality, as shown in Table 3, which was the subgroup analyses stratified by age and BMI. Association between caffeine intake and all-cause mortality were generally similar across subgroups stratified according to the following baseline factors: sex, race, education, income, smoke (yes vs. no), HTN (yes vs. no), diabetes (yes vs. no), and cancer (yes vs. no). The major differences across strata were observed for age and BMI (Table 3). For younger people (20–35 years of age), compared to those with a caffeine intake of <100 mg/day, the HRs for all-cause mortality were higher in the participants with a caffeine intake of 100–200 mg/day (HR, 2.03; 95% CI, 1.03–3.99; *p* = 0.04), and those with a caffeine intake of >200 mg/day (HR, 1.45; 95% CI, 0.74–2.85; *p* = 0.28). For BMI <18.5 kg/m^2^, compared to those with a caffeine intake of <100 mg/day, the HRs for all-cause mortality were higher in the participants with a caffeine intake of >200 mg/day (HR, 1.35; 95% CI, 0.67–2.71; *p* = 0.40).

**Figure 2:**
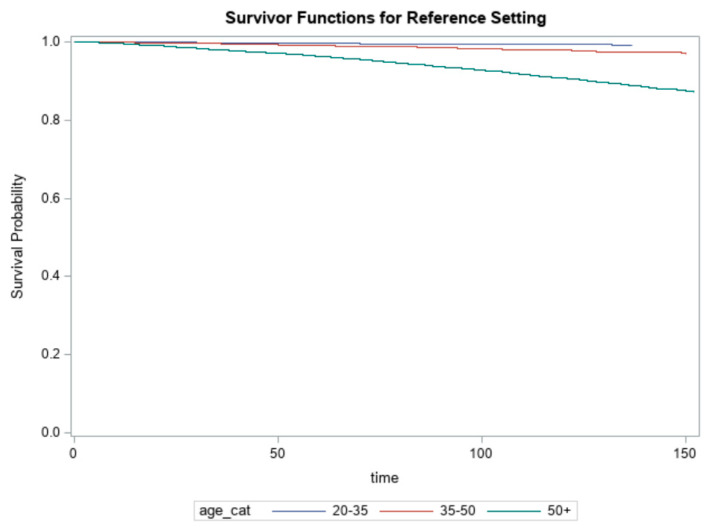
Survival probabilities across age groups.

Figure 2 presented the survival probabilities over time across age groups. As can be seen, the survival probability for the oldest age group was consistently lower than the survival probabilities for the other two younger age groups, which had similar survival probabilities. This graph further confirmed the violation of the proportionality assumption.

In the presence of multiple causes of death, mortality attributable to non-cardiovascular causes (e.g., diabetes or cancer) may be a competing risk for mortality attributable to cardiovascular causes, and we, therefore, calculated cause-specific hazard ratios (Table 2). Accordingly, we censored competing risks for cancer and cardiovascular deaths, respectively. As shown in Table 2, the cause-specific HRs were in general greater than proportional HRs, suggesting weaker inverse associations between caffeine intake and cancer and cardiovascular mortality.

## 4. Discussion

The NHANES 1999–2014 database was used to examine the association between daily caffeine intake and mortality from all-cause, CVD, and cancer after adjusting for age, race, sex, education, income, BMI, smoking status, total daily intake of energy, carbohydrates, protein, and fat, and the presence of diabetes, HTN, and cancer at baseline. Compared to those participants with a caffeine intake of <100 mg/day, those who consumed 100–200 mg/day had a 37% lower risk of CVD death; those who consumed >200 mg/day had a 33% lower risk of CVD death. Compared to those participants with a caffeine intake of <100 mg/day, those who consumed 100–200 mg/day had a 22% lower risk of death; those who consumed >200 mg/day had a 32% lower risk of death. There was no association between caffeine intake and cancer mortality.

Higher caffeine intake was associated with lower CVD mortality in the current study. This finding is consistent with several larger, more recent studies and meta-analyses. In the Alpha Omega Trial [17], compared to those who consumed 0–2 cups (1 cup = 125 mL) of coffee per day, the HRs for CVD mortality was 0.66 (95% CI, 0.52–0.85; *p* = 0.03) for those consuming 2–4 cups of coffee per day, and 0.69 (95% CI, 0.53–0.90; *p* = 0.03) for those consuming >4 cups of coffee per day. In the National Institutes of Health-AARP Diet and Health study [4], compared to those with no coffee intake, the HRs for CVD mortality for people consuming 2–3 cups (cup size not specified) of coffee per day were 0.85 (95% CI, 0.76–0.95; *p* < 0.001) for women, and 0.86 (95% CI, 0.79–0.94; *p* = 0.03) for men. Kim et al. [6] pooled 31 studies including 2,631,398 participants and 81,188 CVD deaths and found an inverse association between coffee intake and CVD mortality with the lowest HR at 0.83 (95% CI, 0.80–0.87; *p* < 0.001) for an intake of 2.5 cups/day compared to those non-coffee drinkers. Crippa et al. [18] pooled 21 studies including 997,464 participants and found an inverse association with CVD mortality with the lowest HR at 0.79 (95% CI, 0.74–0.84; *p* < 0.001) for an intake of 3 cups/day compared with no coffee consumption.

Higher caffeine intake was inversely associated with all-cause mortality in the current study. This finding is consistent with several larger, more recent studies and meta-analyses. In the Alpha Omega Trial [17], compared to those who consumed 0–2 cups of coffee per day, the HRs for all-cause mortality were 0.82 (95% CI, 0.69–0.96; *p* = 0.03) for 2–4 cups/day, and 0.80 (95% CI, 0.67–0.95; *p* = 0.03) for >4 cups/day. In the National Institutes of Health-AARP Diet and Health study [4], compared to those with no coffee intake, the HRs for all-cause mortality for people consuming 2–3 cups of coffee per day were 0.87 (95% CI, 0.83–0.92; *p* < 0.001) for women, and 0.90 (95% CI, 0.86–0.93; *p* < 0.001) for men. Two recent meta-analyses found an inverse association between coffee intake and all-cause mortality, with one of them reporting the lowest HR at 0.85 (95% CI, 0.82–0.89; *p* < 0.001) for an intake of 3.5 cups/day compared to those non-coffee drinkers [6]; the other reporting the lowest HR at 0.84 (95% CI, 0.82–0.87; *p* < 0.001) for an intake of 4 cups/day [18].

The current study found no significant association between caffeine intake and cancer mortality. In previous studies, the findings have been mixed, with some reporting an inverse association [5], while others reporting no association [10,19], and yet another study reporting different associations for men and women [4]. These mixed results may be due to different types of cancer the participants had in each study since coffee seems to be inversely associated with mortality from some cancers but not the others. Researchers reported an inverse association between coffee intake and liver cancer [20] and colon cancer among women [21]. There was no association between coffee intake and breast cancer [22] and gastric cancer [23]. More research is needed to examine the association between coffee/caffeine intake and mortality from different types of cancer.

We conducted subgroup analyses to better understand the potential modification effects. We found that for younger age groups (20–35 years of age), higher caffeine intake was associated with higher all-cause mortality (*p* = 0.04). This finding is consistent with the Aerobics Center Longitudinal Study [24], which reported a positive association between coffee consumption and all-cause mortality among adults below the age of 55 years, although another study [4] reported similar associations across age groups. Future studies focusing on younger age groups are needed to clarify this discrepancy.

Even though not significant, we noticed that for people with a BMI <18.5 kg/m^2^, there was a positive association between caffeine intake and all-cause mortality. One possible explanation is that the metabolic rate of caffeine is significantly higher in lean individuals [25]. Since caffeine seems to be inversely associated with all-cause mortality, a higher metabolic rate of caffeine means having less caffeine in the body in underweight individuals, which may explain this observed positive association, although some other studies have reported similar associations across BMI groups [4,5].

The mechanism of the inverse association between caffeine intake and CVD mortality is not clear. Although caffeine has been considered a risk factor for CVD, a recent animal study suggests that caffeine may protect and repair myocardium through the action of mitochondrial p27, which was known as an inhibitor of the cell cycle [26]. Caffeine also promotes the repair of endothelial function [27] and has anti-inflammatory [28] and bronchodilator effects [29]. Caffeine decreases the risk of depression [30] and has protective effects against some types of cancer [21]. Caffeine helps reduce symptoms of Parkinson’s disease; it also helps with weight control, which will reduce the risk of metabolic syndrome [2]. Although regular caffeine intake increases BP, when ingested through coffee, the pressure effect is small [31]. This pressure effect mainly affects naïve drinkers and only for about 3 h [32]. The half-life of caffeine is 3–7 h [32]. For people with slower caffeine metabolism, coffee increases the risk of HTN, but for people with faster caffeine metabolism, it decreases the risk of HTN [31]. Since HTN is the single largest risk factor for CVD mortality in the US and accounted for 45% of all CVD deaths in 2005 [16], the effects caffeine has on BP might contribute to our understanding of this inverse association between caffeine intake and CVD mortality.

Another possible explanation for the observed inverse association is reverse causality. Participants who have chronic diseases at baseline may decrease or abstain from caffeine consumption. However, some previous studies reported an inverse association between coffee and CVD mortality even when they excluded people with CVD or other chronic diseases at baseline [4,19].

Even though the findings from this study are consistent with some of the larger, more recent studies [4,5,17], the results of earlier smaller studies [10,33] have been highly variable. One of the reasons is that most studies were categorizing participants based on how many cups of coffee they consumed each day [4,5]; only one study categorized participants based on daily caffeine intake (mg/day) [10]. The cup size was not standardized with some studies using 240 mL [5,34], while others using 125 mL [17], 150 mL [19], or 170 mL [35]. Some researchers did not specify how many mL was in a cup [4,33]. Besides, the caffeine content in each cup of coffee varies. In the US, the standard value of caffeine quantity is 96 mg for an 8-oz cup of ground roasted coffee, 64 mg for instant coffee, 48 mg for tea, 30 mg for a 12-oz cola, 64 mg/oz for espresso, and 3 mg for decaffeinated coffee [14]. However, these values were not being used consistently; Greenberg et al. [35] used 159 mg caffeine for each serving of ground roasted coffee. Another reason for the highly variable results from previous studies is that different studies have included different mortality codes, with some of them including stroke death in the CVD mortality rates.

This study had several strengths, including using a nationally representative sample of the US civilian non-institutionalized population (NHANES database 1999–2014). First, it had a large sample size (*n* = 23,878), including both sexes and a wide age range from 20 to 85 years at baseline. Second, it included a multi-ethnic group and a long follow-up period (16 years). Third, it included the most recent NHANES data set, which was also linked to mortality data providing this important clinical outcome measure. Fourth, the NHANES database includes detailed information on many confounding variables allowing for controlling for several known predictors of mortality, such as smoking status, BMI, presence of HTN, diabetes, and cancer at baseline. Finally, this study categorized participants based on how many milligrams of caffeine they consumed daily instead of how many cups of coffee, which is more accurate. These strengths made it possible to perform a robust multivariate Cox proportional hazards regression analysis.

This study had several limitations. First, this is a secondary data analysis using the NHANES database. The data was not collected to answer these specific research questions. We were not involved in the data collection process and had no control over what variables were contained in the dataset. Second, there may be measurement errors because caffeine intake was self-reported. It was collected by one or two 24-h dietary recalls depending on the different NHANES cycles. It was suggested that 24-h dietary recalls underreport the intakes [36], so a comparison between the current study and a representative sample of the US consumers [9] was conducted. For example, for the age group 25–34, the mean caffeine intake for the current study was 166 mg/day, and the mean caffeine intake for Mitchell et al. [9] was 137 mg/day. Third, the amount of caffeine in each cup of coffee is different depending on the preparation methods (espresso, boiled unfiltered, and filtered, etc.) and actual volume in the cup. Since the daily caffeine intake was self-reported, the accuracy of caffeine amount depended on participants’ knowledge of caffeine content and their estimations. Future researchers should quantify caffeine intake more accurately. Fourth, despite efforts to control confounding by a few measured predictors of mortality, the possibility of residual confounding remains. However, the results of the current study reinforced previous larger studies with similar findings. Future studies should consider controlling for physical activity, alcohol intake, menopausal status, red meat, fruit, and vegetable consumption [4]. Physical activity was not adjusted in this analysis because NHANES used inconsistent measurements during the 1999–2014 study period.

## 5. Conclusions

In conclusion, in this large multi-ethnic population, higher caffeine intake was associated with lower CVD and all-cause mortality. Caffeine may have different effects on all-cause mortality among different age and BMI groups. Further research is needed to figure out the mechanism of the inverse association. Even though the current study cannot prove a cause-effect relationship, it provided further evidence for the protective effects of moderate caffeine consumption. The findings of this study support the 2015–2020 US Dietary Guidelines, which suggested that moderate coffee consumption (three to five 8-oz cups/day or up to 400 mg/day of caffeine) can be a part of a healthy diet [14]. Future studies looking at coffee consumption and its health effects should take into consideration of more precise measurement of caffeine like that used in this study than the general convention of cups of coffee per day.

## Figures and Tables

**Table 1:** Baseline Characteristics of Participants Stratified by Daily Caffeine Intake ^a^.

Characteristics	Caffeine Intake (mg/Day)			*p*-Value ^b^
	<100 N (%)	100–200 N (%)	>200 N (%)	
Age				<0.001
20–34	4959 (28.7)	1732 (22.3)	1426 (15.7)	
35–50	4036 (23.4)	1966 (25.3)	2807 (30.9)	
50+	8271 (47.9)	4075 (52.4)	4843 (53.4)	
Sex				<0.001
Male	9477 (54.9)	4009 (51.6)	3879 (42.7)	
Female	7789 (45.1)	3764 (48.4)	5197 (57.3)	
Race				<0.001
White	6174 (35.8)	3967 (51.0)	6348 (69.9)	
Black	4960 (28.7)	1286 (16.5)	768 (8.5)	
Hispanic	4945 (28.6)	2002 (25.8)	1530 (16.9)	
Other	1187 (6.9)	518 (6.7)	430 (4.7)	
Education				<0.001
<High school	5402 (31.3)	2043 (26.3)	1944 (21.4)	
High school	3826 (22.2)	1855 (23.9)	2276 (25.1)	
Some college	3336 (19.4)	1726 (22.3)	2150 (23.7)	
Postgraduate	4678 (27.1)	2138 (27.5)	2698 (29.8)	
Income (dollars/year)				<0.001
<15,000	4826 (29.7)	1865 (25.2)	1986 (22.7)	
15,000–25,000	2065 (12.7)	870 (11.8)	941 (10.8)	
25,000–35,000	2103 (12.9)	967 (13.1)	1026 (11.8)	
35,000–55,000	2713 (16.7)	1332 (18.0)	1611 (18.4)	
55,000–75,000	1609 (9.9)	762 (10.3)	1027 (11.8)	
>75,000	2954 (18.1)	1600 (21.6)	2143 (24.5)	
BMI (kg/m^2^)				<0.001
<18.5	284 (1.7)	122 (1.6)	126 (1.4)	
18.5–24.9	4853 (28.7)	2166 (28.3)	2467 (27.5)	
25–29.9	5644 (33.4)	2658 (34.7)	3202 (35.7)	
≥30	6134 (36.2)	2706 (35.4)	3167 (35.4)	
Smoke				
Yes	6480 (37.6)	3762 (48.4)	5745 (63.3)	<0.001
Hypertension ^c^				
Yes	6115 (35.9)	2726 (35.2)	3082 (34.1)	0.01
Diabetes ^d^				
Yes	2088 (12.3)	913 (12.0)	983 (11.1)	<0.001
Cancer ^e^				
Yes	1491 (8.7)	775 (10.0)	1007 (11.1)	<0.001
Nutrition				
Energy (kcal)	1824 ± 826	2121 ± 901	2294 ± 956	<0.001
Carbohydrate (g)	238 ± 110	266 ± 121	279 ± 131	<0.001
Protein (g)	68 ± 35	80 ± 37	86 ± 39	<0.001
Fat (g)	67 ± 34	78 ± 37	87 ± 40	<0.001

^a^ Data are presented as number (percentage) of participants for categorical variables or mean ± SDs for continuous variables. ^b^ *p*-value was calculated with the chi-square test for categorical variables and ANOVA F-test for continuous variables. ^c^ Defined as either a previous diagnosis of hypertension or intake of antihypertensive medications. ^d^ Defined as either a previous diagnosis of diabetes or an HbA1c level of ≥6.5% or intake of antidiabetic medications including insulin. ^e^ Defined as a previous diagnosis of cancer.

**Table 2:** Hazard Ratios for All-Cause and Cause-Specific Mortality by Daily Caffeine Intake in the National Health and Nutrition Examination Survey (1999–2014).

Mortality Cause	All Participants	Caffeine Intake (mg/Day)		
		<100 (*n* = 12,006)	100–200 (*n* = 5624)	>200 (*n* = 6248)
All-cause mortality				
No. of cases (%)	2206	1150 (52.1)	503 (22.8)	553 (25.1)
Age-adjusted HR (95% CI)		1.0	0.77 (0.69–0.86) *	0.69 (0.63–0.75) *
Multivariable-adjusted HR (95% CI)		1.0	0.78 (0.67–0.91) *	0.68 (0.60–0.78) *
CVD mortality				
No. of cases (%)	394	207 (52.5)	75 (19.1)	112 (28.4)
Age-adjusted HR (95% CI)		1.0	0.70 (0.56–0.88) *	0.72 (0.58–0.89) *
Multivariable-adjusted HR (95% CI)		1.0	0.63 (0.45–0.88) *	0.67 (0.50–0.88) *
Multivariable-adjusted cause-specific HR (95% CI)		1.0	0.67 (0.47–0.94)	0.77 (0.57–1.04)
Cancer mortality				
No. of cases (%)	525	243 (46.3)	115 (21.9)	167 (31.8)
Age-adjusted HR (95% CI)		1.0	0.85 (0.71–1.03)	0.87 (0.74–1.02)
Multivariable-adjusted HR (95% CI)		1.0	0.78 (0.58–1.05)	0.94 (0.72–1.21)
Multivariable-adjusted cause-specific HR (95% CI)		1.0	0.80 (0.59–1.08) *	0.98 (0.76–1.27) *

Abbreviations: HR, hazard ratio; CI, confidence interval. * *p* < 0.05.

**Table 3:** Caffeine Intake and All-Cause Mortality by Age and BMI Groups.

Stratification Variable	Caffeine Intake (mg/Day)				
	<100	100–200			>200
	Referent	HR	95% CI	HR	95% CI
Age					
20–35		2.03	1.03–3.99 *	1.45	0.74–2.85
35–50		0.55	0.37–0.84 *	0.89	0.63–1.24
50+		0.80	0.71–0.89 *	0.67	0.61–0.74 *
BMI					
<18.5		0.76	0.33–1.76	1.35	0.67–2.71
18.5–24.9		0.75	0.62–0.90 *	0.66	0.55–0.80 *
25–29.9		0.87	0.72–1.05	0.66	0.56–0.78 *
≥30		0.77	0.64–0.94 *	0.75	0.63–0.91 *

Abbreviations: BMI: body mass index; HR: hazard ratio; CI: confidence interval. * *p* < 0.05.

## Data Availability

We will make the de-identified data used in this manuscript available to editors upon request.
